# The Road to Creative Achievement: A Latent Variable Model of Ability and Personality Predictors

**DOI:** 10.1002/per.1941

**Published:** 2013-09-02

**Authors:** Emanuel Jauk, Mathias Benedek, Aljoscha C Neubauer

**Affiliations:** Department of Psychology, University of GrazAustria

**Keywords:** creative achievement, creative activities, creative potential, intelligence, structural equation modelling (SEM)

## Abstract

This study investigated the significance of different well-established psychometric indicators of creativity for real-life creative outcomes. Specifically, we tested the effects of creative potential, intelligence, and openness to experiences on everyday creative activities and actual creative achievement. Using a heterogeneous sample of 297 adults, we performed latent multiple regression analyses by means of structural equation modelling. We found openness to experiences and two independent indicators of creative potential, ideational originality and ideational fluency, to predict everyday creative activities. Creative activities, in turn, predicted actual creative achievement. Intelligence was found to predict creative achievement, but not creative activities. Moreover, intelligence moderated the effect of creative activities on creative achievement, suggesting that intelligence may play an important role in transforming creative activities into publically acknowledged creative achievements. This study supports the view of creativity as a multifaceted construct and provides an integrative model illustrating the potential interplay between its different facets.

Creativity is a key concept of human innovation, and much research has been performed to gain a deeper understanding of the creative person. Several perspectives have been used to study creativity, including cognitive, differential, or social psychology approaches (Sternberg & Lubart, 1999), and each paradigm shed light on different aspects of human creativity. A considerable number of different traits that are related to creativity have been identified. Specifically, creative people are thought to be better able to think in different directions (Guilford, 1950), to share certain patterns of personality traits (Feist, 1998, 2010), to be intrinsically motivated (Amabile, 1983), or to have substantial amounts of domain-specific expertise (Simonton, 1999; Weisberg, 2006). Moreover, the relationship between intelligence and creativity has occupied creativity research from the very beginning (Kaufman & Plucker, 2011). Thus, a number of different traits are thought to determine the potential for real-life creative accomplishments, but so far, only few attempts have been made to examine their conjoint influence on actual creative achievement. Therefore, this study aims at investigating the relationship between common psychometric indicators of creativity and real-life creative achievement by means of structural equation modelling.

## The many faces of creativity

Trait creativity in terms of psychometrically tested ability can generally be distinguished from real-life *creative achievement* (Eysenck, 1993, 1995). The former concept is hereby often labelled *creative potential*, highlighting that it reflects rather a predictor of real-life creativity than creativity *per se* (Runco & Acar, 2012). In line with this distinction, creativity research used to sort into two poles, focusing either on the study of psychometrically tested creative potential or on eminent real-life creativity (also called ‘little-C’ vs ‘big-C’; cf. Kaufman & Beghetto, 2009). Besides these two approaches, a complementary line of research has emerged that is concerned with real-life creativity within the general population, referred to as *everyday creativity* (Richards, 1993, 2010). As outlined later, everyday creativity can be considered a behavioural prerequisite of actual creative achievement.

### Creative potential

Creative potential refers to an individual's cognitive ability to generate something novel and useful (Barron, 1955; Runco & Jaeger, 2012; Stein, 1953) and reflects a normally distributed trait (Eysenck, 1995). It is commonly assessed by means of divergent thinking (DT) tests (Runco, 2010) such as the Torrance Tests of Creative Thinking (Torrance, 1966), the Guilford tests (Wilson et al., 1953), or the Wallach and Kogan tests (Wallach & Kogan, 1965). DT tests typically involve ill-structured problems for which a variety of possible solutions can be found. In the alternate uses (AU) task, a popular example of a DT task, participants are instructed to find many different creative uses for everyday objects in a given time (for example, brick – ‘use for karate demonstration’). The performance in DT tests can be scored with respect to different criteria usually involving ideational fluency, that is, the quantity of ideas, and/or originality, that is, the quality of ideas. Whereas ideational fluency reflects the number of ideas in a given time, ideational originality is commonly assessed by means of creativity evaluations of the generated ideas. Recent works using subjective top scoring or snapshot scoring (Benedek et al., [Bibr b13]; Silvia, Martin, & Nusbaum, 2009; Silvia et al., 2008) found that fluency and originality show discriminant validity and relate differently to other cognitive functions: Ideational fluency draws upon executive functioning, whereas originality is related more strongly to intelligence (Benedek et al., 2012).

### Everyday creativity

The concept of everyday creativity emerged from the study of real-life creative activities within the general (noneminent) population. Everyday creativity is ‘defined in terms of human originality at work and leisure across the diverse activities of everyday life’ (Richards, 2010, p. 190). Popular measures of everyday creativity are the revised Creative Behavior Inventory (CBI; Dollinger, 2003; based on the original CBI by Hocevar, 1979) and the Biographical Inventory of Creative Behavior (BICB; Batey, 2007) (for a review, see Silvia et al., 2012). Both inventories contain lists of various creative activities (e.g. ‘drew picture/cartoon’ or ‘designed costume/textile’) and assess the number of activities performed within a given period. Everyday creativity, assessed by means of biographical measures, was found to be normally distributed just like creative potential (Richards et al., 1988). Thus, an important implication of this concept is that everyone can be involved in creative activities to a varying extent, or a varying degree of absorption. Everyday creativity is considered a behavioural prerequisite of actual creative achievement, or as Richards (2010) put it: ‘Everyday creativity can be seen as the ground from which (a later and) more publicly celebrated accomplishment can grow’ (p. 193).

### Creative achievement

Creative achievement refers to actual real-life creative accomplishments (such as composing a piece of music, making a scientific discovery, or writing a book; cf. Carson et al., 2005) and is commonly assessed by means of biographical measures. Creative achievement is supposed to follow a highly skewed distribution (Eysenck, 1995), or even a Lotka curve (Simonton, 1999), with the vast majority of the population displaying low levels of creative achievement and only few who attain eminent, high-level creative achievement. A popular measure of creative achievement is the Creative Achievement Questionnaire (CAQ; Carson et al., 2005). The CAQ asks for the attained level of achievement in 10 different domains [for instance, in the domain of music, the levels of achievement range from *I have no training or recognized talent in this area* (0 points) to *My compositions have been critiqued in a national publication* (7 points)]. Thus, the CAQ measures primarily qualitative, not quantitative, aspects of creative achievement. The CAQ was found to successfully differentiate between artistic and nonartistic students (Vellante et al., 2011). Discriminant validity evidence comes from the finding that the CAQ is not related to general academic achievement (Hirsh & Peterson, 2008).

## Correlates of real-life creativity

Dealing first with everyday creativity, the CBI was found to correlate with openness to experiences and tests of creative potential (Dollinger, 2007) as well as self-rated creativity (Wigert et al., 2012). The BICB was found to be related to openness and extraversion (Batey et al., 2010; Furnham & Bachtiar, 2008; Furnham et al., 2008) and facets of schizotypy (Batey & Furnham, 2008). No significant influence of intelligence on the BICB was found across several studies (Batey et al., 2010; Furnham & Bachtiar, 2008; Furnham et al., 2008). As the authors conclude, ‘everyday creative accomplishment is not reliant upon intellect’ (Batey et al., 2010, p. 535).

The picture looks differently for creative achievement: In an early study, King, Walker, and Broyles (1996) instructed participants to freely list their creative accomplishments and subjected these lists to peer ratings, thus considering not only quantitative but also qualitative aspects of real-life creativity. They found that the quality ratings were best predicted by an interaction between creative potential and openness to experiences; that is, high creative potential and high openness. Moreover, quality ratings were significantly related to intelligence (*r* = .27). Further studies involving the CAQ found significant correlations with openness to experiences (Silvia, Kaufmann & Pretz, 2009; Silvia et al., 2012) and with intelligence (Carson et al., 2003; Kéri, 2011). A recent meta-analysis estimated the relationship of creative achievement with creative potential as *r* = .22 and that of creative achievement with intelligence as *r* = .17 (Kim, 2008).

It hence can be concluded that openness is a consistent correlate of both everyday creative activities and creative achievement, whereas intelligence is exclusively related to actual creative achievement. Moreover, it is interesting to note that creative achievement displays a highly skewed distribution (Silvia et al., 2012), whereas creative activities and most other relevant traits do not. As noted by several researchers (e.g. Eysenck, 1995; Simonton, 1999), skewed distributions are likely to arise from a multiplicative, synergistic, rather than additive interplay of various factors. This suggests that several traits have to appear in combination in order to allow for high creative achievement. Although the involvement in everyday creative activities can be considered a necessary condition for creative achievement (i.e. it is hardly possible to make a significant contribution to a field without being regularly engaged in it), relevant cognitive and noncognitive factors may determine the actual and attainable level of achievement.

## The role of intelligence in creative achievement

We propose that intelligence plays a central role in creative achievement for two reasons. First, there is a robust relationship between intelligence and ideational originality, thus pointing to common cognitive mechanisms underlying divergent and convergent thinking processes (Benedek et al., 2012; Cropley, 2006; Nusbaum & Silvia, 2011a). Second, creative achievements generally represent extensive, complex tasks that may draw upon intelligence: Putting creative ideas into practice usually requires a good deal of planning and elaboration in the long run, and although intelligence-related demands may differ between creative domains (Getzels & Csikszentmihalyi, 1972), being of higher intelligence will generally not be detrimental to creative endeavours. It is well known from other fields of research that intelligence is a central predictor of general career success (cf. Schmidt & Hunter, 2004).

In line with these considerations, intelligence was found to predict creative achievement in longitudinal studies: Plucker's (1999) reanalysis of longitudinal data originally collected by Torrance (1972) found latent factors of both intelligence and creative potential to be predictive of real-life achievements as much as 20 years later. Creative potential was the strongest predictor of creative achievement, but intelligence explained incremental variance over and above creative potential. Further studies dealing with Torrance's data reported that creative potential and intelligence were significant predictors of creative achievement in a 40-year follow-up (Cramond et al., 2005). In a 50-year follow-up, Runco et al. (2010) found that creative potential was associated with *personal* achievements (which can be considered everyday creative activities), whereas *publicly acknowledged* creative achievement was related to an interaction between intelligence and creative potential. Wai et al. (2005) found Scholastic Aptitude Test scores within a gifted group of 13-year-olds to predict creative achievement 20 years later. Although measures of creative potential were not used, this study vividly demonstrates that intelligence is an important factor even within high-ability groups. In a 45-year longitudinal study, Feist and Barron (2003) found that intelligence at age 27 predicts lifetime creative achievement at age 72. Additionally, personality traits such as self-confidence and openness explained incremental variance over and above intelligence.

Another prominent concept regarding the interplay between intelligence and creativity is the threshold hypothesis. It assumes that intelligence fosters creativity only up to a threshold of about 120 IQ points and thereafter loses its impact. The threshold hypothesis is commonly investigated using measures of creative potential (cf. Kaufman & Plucker, 2011). Although a meta-analysis of the previous literature yielded no evidence for a threshold effect (Kim, 2005), recent studies using new methodology found partial support for the threshold hypothesis (Jauk et al., 2013; Karwowski & Gralewski, 2013). Specifically, our recent results suggest that the intelligence threshold depends on the employed criterion: Although we observed threshold effects for indicators of creative potential, we found no evidence for an intelligence threshold in creative achievement. Thus, intelligence may foster creative achievement throughout the whole ability range.

## The present research

This study aims to investigate the influence of common psychometric indicators of creativity (including openness, ideational fluency, ideational originality, and intelligence) on everyday creative activities and actual creative achievement. Because the involved constructs are known to share substantial amounts of variance and may display complex interdependencies, we set up latent variable structural equation models to avoid the typical fallacies of correlational research (cf. Silvia, 2008). Because the existing measures of everyday creativity and creative achievement differ substantially with respect to the included domains of creativity, we used a newly devised inventory that captures both constructs in a standardized way across the same major domains of real-life creativity.

On the basis of the findings presented earlier, we hypothesized that both creative activities and creative achievement are predicted by openness to experiences and creative potential. Moreover, we expect that everyday creative activities predict actual creative achievement. This assumption has strong face validity but has to our knowledge not yet been tested empirically. Finally, we assume that intelligence predicts creative achievement but not creative activities. Because creative achievement is thought to depend upon several factors that have to appear in combination, we also tested whether intelligence actually moderates the influence of creative activities on creative achievement.

## METHOD

### Participants

Participants were recruited via a local free newspaper as well as the university's mailing lists. We included people with an age between 18 and 55 years, German as the mother tongue, and the absence of any mental or neurological disorders. After excluding one person because of excessive missing data, the final sample consisted of *N* = 297 participants (101 men) who took part in a larger study on cognitive ability, motivation, and personality (also Jauk et al., 2013). Participants were on average 30 years old (*SD* = 10.68). Of the participants, 16% had 9 years of schooling, 60% had 12 years of schooling, and 24% had a university degree. Participants were paid for taking part in the study.

### Measures

#### Creative potential

Creative potential was measured by means of three AU tasks and three instances (IN) tasks. In the AU tasks, participants were required to find as many novel and uncommon uses as possible for a *can*, a *knife*, and a *hairdryer*. In the IN tasks, participants were instructed to figure out many novel and uncommon solutions to the problems ‘What can make noise’, ‘What can be elastic’, and ‘What could one use for quicker locomotion’? The tasks were administered on a PC, and participants were required to enter their ideas via a keyboard. Each task lasted for 2 minutes. Ideational fluency was defined as the number of ideas given in the task. Ideational originality was assessed by means of subjective top 3 scoring, which reflects the rated creativity of the three best ideas within each task (Benedek et al., in press[Bibr b13]). To this end, participants were asked to identify their most creative ideas, and the three best ideas were then rated for creativity. Ratings were performed by four raters (three women) experienced in DT assessment on a 4-point scale ranging from 1 (*not creative*) to 4 (*very creative*). Mean interrater reliabilities were ICC = 0.80 in the AU tasks and ICC = 0.69 in the IN tasks. Ratings were then averaged across raters. The subjective top-scoring method was shown to overcome the confounding of ideational originality and fluency while concurrently providing highly reliable and valid scores (Benedek et al., in press[Bibr b13]; Silvia et al., 2008). Total scores of ideational fluency and originality were computed by averaging across the six DT tasks.

#### Creative activities and creative achievement

In order to assess participant's everyday creative activities and actual creative achievements, we administered a newly devised inventory of creative activities and achievements (ICAA).^1^ This inventory assesses creative activities and achievements in eight domains, including literature, music, arts and crafts, creative cooking, sports, visual arts, performing arts, and science and engineering. Rather than using existing measures such as the BICB and the CAQ, the use of the ICAA appeared necessary to ensure that activity and achievement scores refer to the same domains of creative accomplishment. The ICAA was piloted in a sample of about 350 people and further validated in smaller samples of art students.

The *activities scale* of the ICAA was constructed in the style of existing scales that measure everyday creativity such as the revised CBI (Dollinger, 2003; Hocevar, 1979) or the BICB (Batey, 2007). Participants report on a 5-point scale how often they carried out certain activities within the last 10 years (*never*, *one to two times*, *three to five times*, *5–10 times*, and *more than 10 times*). Responses are assigned 0–4 points. The ICAA includes six relevant activities for each of the eight domains, ensuring equal representation of all domains. For example, in the literature domain, participants are presented with statements such as ‘wrote a short literary work (e.g., poem, short story)’ or ‘wrote a blog entry’. Domain scores are obtained by summing points across domain-specific activities, and a total score can be computed by further summing across domains.

The *achievement scale* of the ICAA is conceptually similar to the CAQ by Carson et al. (2005). Participants are asked to indicate which achievements they have already attained in each of the eight domains. The achievements range from *I have never been engaged in this domain* (0 points) to *I have already sold some of my work in this domain* (10 points). As in the CAQ, all applying levels can be checked, but in contrast to the CAQ, no extra points are given for repeatedly attaining certain achievements (e.g. selling one's work twice or three times).

In addition to the ICAA, we administered a German translation of the CAQ to obtain evidence of convergent validity for the ICAA achievements scale. Moreover, we asked participants to freely list their creative achievements (similar to King et al., 1996) in order to check for relevant achievements in domains other than those included in the ICAA and the CAQ.

#### Intelligence

General intelligence (*g*) was assessed by means of four subtests of the Intelligence Structure Battery (Intelligenz-Struktur-Batterie, INSBAT; Arendasy et al., 2004), which is theoretically grounded on the Cattell–Horn–Carroll model of intelligence (for an overview, see McGrew, 2009). Four computer-based tests were selected to reflect a broad representation of *g* including *figural inductive reasoning* (figural induktives Denken), *verbal short-term memory* (verbales Kurzzeitgedächtnis), *arithmetic flexibility* (arithmetische Flexibilität), and *word meaning* (Wortbedeutung). Detailed descriptions of the single tests are given in Jauk et al. (2013).

The INSBAT is based on item response theory and allows for tailored testing. Target reliability for each scale was set to α = .60, which results in an average of 10 items per test, or an average duration of 10 minutes per test.

#### Openness to experiences

We assessed openness to experiences by means of the Big Five Structure Inventory (Big-Five Struktur Inventar; Arendasy et al., 2011). The test is based on item response theory and was shown to have good correlations with the German Big Five questionnaire NEO-PI-R, while internal consistency is even higher (Arendasy et al., 2011). Openness was assessed by means of six facets (openness to fantasy, aesthetics, feelings, actions, ideas, and norms/values) with 10 items each, thus resulting in a total number of 60 items for the factor. The test was administered without time restriction.

### Procedure

The experiment took place in a computer laboratory. Groups of up to 10 people performed all tests on standard desktop computers. Two experimenters informed participants about the purpose of the study and were present during the experimental session. Because this study was part of a larger screening for further investigations, participants also completed motivation scales and a speed of information processing task. The order of tasks was as follows: After completing a sociodemographic questionnaire and motivation scales, participants performed the INSBAT for about 50 minutes followed by a break of 15 minutes. Next, participants worked on the speed of information processing task, the tasks of creative potential, the ICAA, the CAQ, and finally the Big Five Structure Inventory. The total test session took about 2.5 hours. The procedure was approved by the Ethics Committee of the University of Graz.

## RESULTS

### Descriptive statistics and intercorrelations

**Figure 1:**
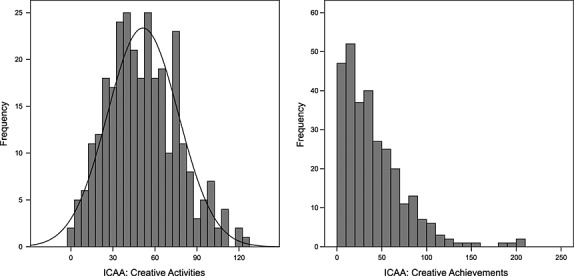
Frequency distributions of the Inventory of Creative Activities and Achievements (ICAA) scales.

Figure 1 shows the distributions of the ICAA activities and achievements scores. As predicted by theory, the achievements scale displayed positive skewness (skewness = 1.78, *p* < .01; kurtosis = 4.66, *p* < .01), whereas the activities scale was normally distributed (skewness = 0.38, *ns*; kurtosis = −0.23, *ns*). Measures of creative potential, openness to experiences, and intelligence were all normally distributed. Internal consistency of the ICAA was assessed by means of Cronbach's α across the eight scales of the inventory. Coefficients were α = .78 for the activities scale and α = .71 for the achievements scale.

Table 1 displays the descriptive statistics and intercorrelations of the observed variables. Measures of real-life creativity (activities and achievements) showed significant positive correlations with all other variables including openness, ideational fluency, and originality, as well as intelligence. The highest correlations for the creative activities scale were found with the creative achievement measures, and with openness. The ICAA achievement scale was highly correlated with the CAQ (*r* = .68; *r*_S_ = .70) supporting its convergent validity. Moreover, a substantial positive correlation was observed between intelligence and originality (Table 1). The latent relationships between these constructs are presented in the following section.

**Table 1:** Descriptive statistics and intercorrelations of the employed measures

	Min	Max	*M* (*SD*)	2	3	4	5	6	7
ICAA: activities (1)	0.00	124	51.42 (25.36)	0.66	0.52	0.13	0.49	0.32	0.28
ICAA: achievements (2)	0.00	208	40.72 (35.15)		0.68	0.27	0.37	0.28	0.27
CAQ total (3)	0.00	151	12.97 (16.03)			0.17	0.29	0.39	0.13
Intelligence (4)	−4.33	2.40	−0.56 (1.10)				0.14	0.20	0.40
Openness (5)	−2.07	2.03	0.16 (0.78)					0.26	0.19
CP: fluency (6)	4.17	27.17	12.37 (3.89)						0.18
CP: originality (7)	1.35	2.47	2.02 (0.18)						

*Note*: *N* = 297. Intelligence and openness scores reflect person parameters according to the item response theory model. Intelligence scores are based on the average of the subtests figural inductive reasoning, verbal short-term memory, and arithmetic flexibility (for details, see Method section).

ICAA, Inventory of Creative Activities and Achievements; CAQ, Creative Achievement Questionnaire; CP, creative potential.

Correlation coefficients above *r* = .11 are significant at *p* = .05, coefficients exceeding *r* = .15 are significant at *p* = .01.

### Model specification

A structural equation model was established in order to analyse the latent relationships between real-life creativity measures (creative activities and creative achievements), creative potential (ideational fluency and originality), openness to experiences, and intelligence. Model estimation was performed with Mplus 5.2 using the maximum likelihood procedure with robust standard errors (MLR) in order to account for non-normality in the data. All regression coefficients were standardized. We followed a two-step modelling approach (Anderson & Gerbing, 1988) in which identified parts of the measurement models were evaluated separately before testing the structural relationships among the latent constructs. This procedure ensures that the latent constructs are adequately measured before examining their structural relationships. Modifications that were made to the measurement models are described in the following section. Parameter estimates of the measurement models are not presented separately because they virtually equalled the results of the final model.

#### Measurement models

**Figure 2:**
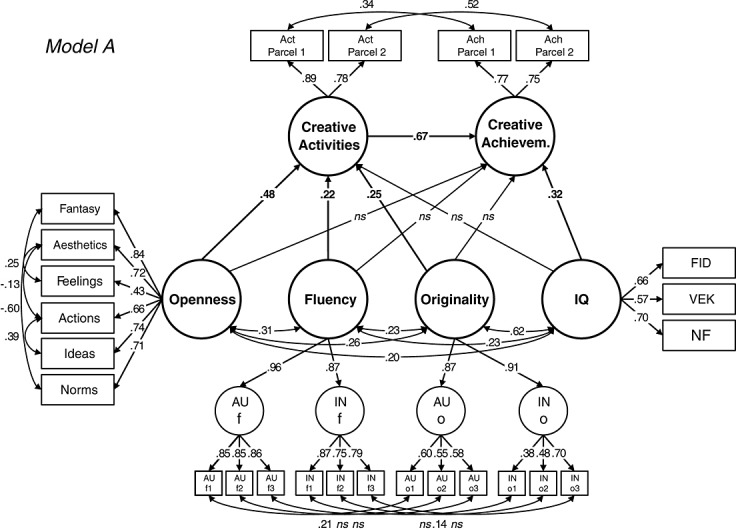
Structural equation model for the prediction of creative activities and achievements. Error terms are not displayed. Detailed explanations and abbreviations are given in the Results section.

In each measurement model, the first indicator (left to right or top to bottom in Figure 2) of each latent variable was fixed to 1. The measurement models of creative potential consisted of two separate two-factor hierarchical confirmatory factor analysis (CFA) models for ideational fluency and ideational originality as depicted in Figure 2. Each score was defined by the two DT task types (i.e. fluency was measured by AUf and INf and originality by AUo and INo), which again were defined by the three tasks per task type (e.g. AUf1, AUf2, and AUf3). Because fluency and originality reflect two different indicators that were assessed using the same six tasks, error correlations were free to vary between each pair of indicators in order to account for task-specific variance (e.g. the error term of AUf1 was allowed to correlate with the error of AUo1). For the measurement of openness to experiences, the six facets were regressed onto a single latent factor. Four error correlations were specified between the six facets (Figure 2) in order to reach adequate fit of the measurement model. It is well known that CFA models of the Big Five have to deal with cross-loadings and error correlations in order to reach acceptable fit to the data (cf. Marsh et al., 2010). For the measurement of general intelligence, the four subtests were regressed onto one latent factor. The subscale *word meaning* was excluded from further calculations because variance explained in the observed variable was not significant (*R*^2^ = 6%, *p* = .06).

Creative activities and achievement, as assessed by the ICAA, were each defined by two item parcels that comprised the first four and second four domain scales of the inventory (i.e. literature, music, handicraft, and creative cooking vs sports, visual arts, performing arts, and science and engineering, respectively). Similar to the measurement model of creative potential, error correlations between the corresponding parcels were allowed in order to account for unique (domain-specific) variance. Despite the fact that each domain has its unique portion of variance, this procedure allowed us to investigate domain-general variance inherent to creative activities and achievements. Although some concerns have been expressed towards the use of parcels, it can well be justified when the goal of a study is to understand latent structure of a set of constructs rather than the structure of a set of items (Little et al., 2002).

#### Structural model

The effects of the predictor variables openness to experiences, ideational fluency, ideational originality, and intelligence were tested on creative activities and achievements in the structural part of the model. Thus, all paths were free to vary and, although we did not expect intelligence to influence creative activities, we did not rule out this possibility *a priori*. Moreover, creative activities were assumed to influence creative achievement. Correlations between openness, ideational fluency, originality, and intelligence were all free to vary as these constructs are known to be correlated.

### Latent variable model results

We assessed model fit using the χ^2^ test, the comparative fit index (CFI), the root mean square error of approximation (RMSEA), and the standardized root mean square residual (SRMR) (Beauducel & Wittmann, 2005; Hu & Bentler, 1998, 1999).

The model estimation converged to an admissible solution. Although we obtained a significant χ^2^ test, all other indices indicate good fit (χ^2^(244) = 348.82, *p* < .01; CFI = 0.965; RMSEA = 0.038; 90% CI [0.029, 0.047], *p*_RMSEA<0.05_ = .99; SRMR = 0.052). Because sensitivity of the χ^2^ test is known to increase with sample size, a common practice is to evaluate model fit by χ^2^/*df*. This model showed a χ^2^/*df* of 1.43, which is below the commonly employed criterion of 2 indicating good model fit (Byrne, 1989, p. 55).

#### The prediction of creative activities and creative achievement

All correlations between the four predictor variables openness, ideational fluency and originality, and intelligence were statistically significant. Openness to experiences showed moderate correlations with all other variables. Moreover, latent factors of ideational originality and fluency were moderately correlated. There was a strong association between ideational originality and intelligence, whereas the correlation between fluency and intelligence was markedly lower (Figure 2).

The results of the structural regression model indicate that openness to experiences is the strongest predictor of creative activities (β = .48, *p* < .001). In addition, ideational fluency (β = .22, *p* < .01) and ideational originality (β = .25, *p* < .05) were significant predictors of creative activities, but intelligence was not (β = −.13, *p* = .23). The overall variance explained in the latent variable creative activities was *R*^2^ = 44%. Turning to the prediction of creative achievement, we find that the contrary holds true: Here, neither openness nor the indicators of creative potential had significant effects. Instead, creative achievement is predicted by creative activities (β = .67, *p* < .001) and intelligence (β = .32, *p* < .001). Openness, ideational fluency, and ideational originality had no significant effects (openness: β = .05, *p* = .45; fluency: β = .04, *p* = .62; originality: β = −.12, *p* = .23). The predictors accounted for *R*^2^ = 60% of the variance in the latent variable.

#### Indirect effects on creative achievement

We also investigated whether openness and creative potential have an indirect influence on creative achievement, that is, if creative activities mediate their effects on creative achievement. We found that openness had a significant indirect effect on creative achievement (β = .32, *p* < .001). Moreover, the indirect effects of ideational fluency (β = .15, *p* < .01) and originality (β = .17, *p* < .05) were significant, too. Thus, the effects of all three variables on creative achievement are fully mediated by creative activities.

#### Intelligence as a moderator variable

In order to test our hypothesis that it takes intelligence to convert creative activities into creative achievements, we examined if intelligence moderates the influence of creative activities on creative achievement. Thus, from the model presented earlier (model *A*), we set up a moderation model *A*′ that involved a latent interaction term between intelligence and creative activities.^2^ The resulting model converged to an admissible solution and showed a good fit (χ^2^(393) = 468.64, *p* < .01; χ^2^/*df* = 1.19; CFI = 0.979; RMSEA = 0.025; 90% CI [0.015, 0.034], *p*_RMSEA<0.05_ = 1.00; SRMR = 0.051). Factor loadings of the indicators of the latent interaction variable ranged from 0.42 to 0.81.

**Figure 3:**
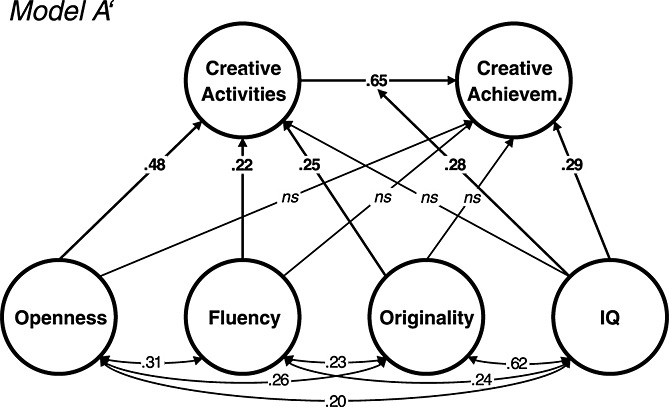
Structural part of the latent moderation model.

We found a significant interaction between intelligence and creative activities on creative achievement (β = .28, *p* < .001) while all other parameter estimates remained virtually unaffected (Figure 3). Thus, the influence of creative activities on creative achievement is moderated by intelligence. The total variance explained in creative achievement was *R*^2^ = 67%.

A comparison of the moderation model *A*′ with the initial model *A* by means of the Akaike information criterion (AIC) shows that the initial model should be preferred over the moderation model (AIC*_A_*: 32 351.14, AIC_*A*′_: 46 933.88). Nonetheless, the AIC is a parsimony-adjusted index that punishes a higher number of free parameters (which are needed to establish the latent interaction), and yet little is known concerning the comparison of models with and without latent interaction variables established by means of the residual-centring approach. The indirect effects of openness and creative potential were also significant in the moderation model *A*′ (openness: β = .31, *p* < .001; Ideational fluency: β = .14, *p* < .01; Ideational originality: β = .16, *p* < .05). Thus, the indirect effects are moderated by intelligence in terms of moderated mediation.

## DISCUSSION

The aim of this study was to disentangle several aspects of the multifaceted construct of creativity: We tested to which extent real-life creativity, in terms of everyday creative activities and actual creative achievement, is determined by creative potential, openness to experiences, and intelligence. The results indicate that these cognitive and noncognitive traits play different roles for the realization of creative activities and creative achievement.

Everyday creative activities include minor creative accomplishments (e.g. drawing a picture or writing a piece of music) representing personal achievements, whereas creative achievement usually refers to publically acknowledged achievements (Kaufman & Beghetto, 2009; Runco et al., 2010). Although the extent of engagement in creative activities depends upon openness to experiences and creative potential (i.e. the ability to produce original ideas and to produce many of them), actual creative achievement, in turn, depends conjointly on intelligence and the amount of creative activities.

These findings are well in accordance with the literature suggesting that everyday creative activities are to be distinguished from actual creative achievement (Kaufman & Beghetto, 2009). Our results further suggest that everyday creativity and creative achievement should not be viewed as competing but rather as complementary concepts. Everyday creative activities can help to bridge a gap from creative potential, in terms of tested ability, to actual creative achievement: We found that openness and creative potential exert their influence on creative achievement via the path of creative activities. People high in openness and high in creative potential engage more frequently in creative activities (and perform minor/personal creative accomplishments), but intelligence may determine whether these remain at the level of personal accomplishments or may become publically acknowledged achievements.

### Openness and creative potential foster creative activities

Openness to experiences was the strongest predictor of creative activities. This finding is well in line with the literature suggesting that openness is a consistent and significant correlate of various aspects of creativity (Batey & Furnham, 2006; Feist, 1998, 2010; Nusbaum & Silvia, 2011a), especially everyday creativity (Silvia, Nusbaum, Berg, Martin, & O'Conner, 2009). Open people are curious, have a need for variety, and actively seek out new activities (McCrea and Costa, 1997), which may lead them to engage in different everyday creative activities. Moreover, openness is thought to reflect an ‘investment trait’ relevant to creativity (Chamorro-Premuzic & Furnham, 2005) and fosters the acquisition of a broader general knowledge (Cho et al., 2010). Taken together, open people may engage in creative activities more frequently and thereby acquire a basis of experience and knowledge upon which possible later achievements can build.

Creative potential indicators, ideational fluency and originality, both had incremental effects on the amount of creative activities over and above openness to experiences. Everyday creative activities such as interpreting a piece of music, making up a recipe, or designing a website hence could be considered open-ended problem situations that depend on the ability to come up with a variety of original ideas. Our findings further support the view that ideational fluency and ideational originality are discriminable aspects of creative potential. Both indicators predicted unique variance in creative activities. Moreover, although fluency was related more strongly to openness to experiences, we observed a strong correlation between originality and intelligence. This is in line with recent research demonstrating that the qualitative measure of ideational originality draws strongly upon intelligence, whereas ideational fluency, as a purely quantitative indicator, rather depends upon executive functioning (Benedek et al., 2012; Gilhooly et al., 2007; Nusbaum & Silvia, 2011b). Concerning intelligence, our recent analyses pointed to a nonlinear relationship between intelligence and ideational originality: Originality may strongly draw upon intelligence in low-intelligence to average-intelligence individuals, whereas other factors might determine the originality of brighter people's ideas (Jauk et al., 2013). In summary, openness can be viewed as a personality requisite of creative activities, whereas ideational fluency and originality can be considered cognitive requirements that are relevant to the performance of creative activities.

### The role of intelligence in creative achievement

The finding that intelligence is important for creative achievement supports the view that successful creators are not solely creative but also bright (Kéri, 2011; Sternberg & Lubart, 1996; Sternberg & O'Hara, 1999). This notion is well in line with previous research suggesting that intelligence is crucial for all kinds of complex problem solving and may especially be needed to put creative ideas into action (Sternberg & O'Hara, 1999). Although intelligence and creative activities contribute additively to creative achievement, results from the moderation model suggest that the interplay of both factors can explain additional variance in creative achievement. Moreover, because creative activities mediate the influence of openness and creative potential on creative achievement, this model actually reflects a moderated mediation. These findings are in accordance with the notion that skewed distributions, repeatedly observed for measures of creative achievement, arise by means of interactional effects (Eysenck, 1995; Simonton, 1999). That is, only people that are both highly intelligent and performing many creative activities (supported by their openness and creative potential) may reach high creative achievement. Finally, these results corroborate the observation that intelligence generally does not predict the exertion of creative activities (although latent factors of intelligence and ideational originality displayed a large amount of shared variance) but is particularly relevant to creative achievement (e.g. Batey et al., 2010).

### A multifactorial model of creative achievement

Creative achievement is generally believed to be determined by several cognitive and noncognitive factors (Eysenck, 1995). Specifically, certain combinations of relevant traits are thought to form the basis for actual creative achievement (Simonton, 1999). Although openness and creative potential had no direct effects on creative achievement in our latent variable model, they did have indirect effects, mediated by creative activities. That is, openness and creative potential may not directly increase creative achievement, but they foster the exertion of creative activities. Specifically, openness may lower the *behavioural threshold* for engaging in creative activities (Feist & Barron, 2003). That is, when given the chance to engage in a creative activity, an open person is more likely to actually seize this opportunity. Creative potential, in terms of the ability to come up with many novel ideas, may then facilitate the exertion of creative activities and lead to encouraging experiences, which, in turn, may result in sustained engagement in a certain field. By this means, a person may acquire a broad base of experience and knowledge in a certain domain. Finally, it may depend on intelligence to which extent one can convert the acquired skills or expertise into notable creative achievements (Sternberg & Lubart, 1996).

### Limitations and directions for future research

A key assumption to the latent variable models presented in this study is that creative achievement is causally preceded by creative activities. In our view, this assumption has strong face validity (e.g. it is hardly possible to win a prize for a musical composition without being engaged in the field of music). Nonetheless, the structural equation models presented here are not capable of testing this assumption. Only a longitudinal study could clarify this issue empirically. Moreover, it has to be noted that the influence of creative activities on creative achievement is likely to be overestimated because of shared method variance (assessment within the same inventory). Future studies could use different indicators of creative activities and creative achievement to address this issue.

Several scholars have stressed the importance of expertise on creative achievement (e.g. Simonton, 1994, 2004; Weisberg, 2006). Because we did not directly assess the amount of time or engagement that a person devoted to a certain field, we had only limited possibilities to explore these relationships. We did, however, take up participant's age as a predictor variable in the structural equation models. Age did not have significant effects on creative activities or creative achievement. Contrary to what could be expected, age displayed negative zero-order correlations with creative achievements (*r* = −.23) and creative activities (*r* = −.12). Thus, these effects are most likely to reflect cohort differences (older generations may have had less favourable conditions for creative endeavours).

Closely related to the acquisition of expertise is the importance of motivational variables in creative achievement (e.g. Amabile, 1983). Creative achievement usually requires a good deal of hard work, and motivation can be considered an important prerequisite in order to stick to a certain task over a longer period. Especially intrinsic motivation is assumed to be closely related to real-life creativity (Hennessey, 2003, 2010). The assessment of intrinsic motivation, however, makes sense only with regard to a certain domain or activity (e.g. a person may be intrinsically motivated in the field of music, but not in science). Although this study employed a domain-general approach, future studies exploring domain-specific models of real life could examine potential additional effects of intrinsic motivation. In this context, it was also noted that intelligence-related demands may vary between different fields of creative endeavour (Getzels & Csikszentmihalyi, 1972). For instance, achievement in the field of music was found to be more strongly related to intelligence than creative potential as compared with other domains (Kim, 2008).

## CONCLUSIONS

This study investigated the relationships between different aspects of the multifaceted construct of creativity and specifically aimed at linking real-life creativity with common indicators of trait creativity. We examined a model that traces creativity from its cognitive components over everyday creative activities to actual creative achievement. We found that the exertion of everyday creative activities depends upon openness to experiences and creative potential. Turning creative activities into actual achievements, in contrast, depends on intelligence. Thus, this study integrated several lines of research, resulting in a more comprehensive picture of the interplay between relevant determinants of creative achievement.

## Footnotes

1 The ICAA is available in English and German and can be obtained from the second author upon request.

2 Moderation analyses with continuous latent variables follow the same principle as originally described for manifest variables by Baron and Kenny (1986): If *M* moderates the influence of *X* on *Y*, the product term *M* * *X* should explain variance in *Y* over and above the main effects of *X* and *M* (also Little et al., 2007). Latent product terms are set up by multiplying each combination of the single indicators of *X* and *M*. Collinearity between *M* * *X*, *X*, and *M* must be avoided by means of orthogonalization. We set up a moderation model for the latent variables creative activities (*X*) and intelligence (*M*) and tested their conjoint influence on creative achievement (*Y*). Orthogonalization was performed by means of the residual-centring approach as described by Little et al. (2006). This involves the following: (i) building the product terms between each pair of indicators of *X* and *M*; (ii) regressing each of these product terms onto all indicators in a standard multiple regression model; and then (iii) using the residuals of each regression analysis (i.e. the proportion of variance in the interaction term that cannot be explained by a linear combination of its constituent terms) as latent variable indicators. Given two indicators of creative activities and three indicators of intelligence, we used six regression residuals as indicators of the latent interaction variable. Error correlations were allowed between each pair of indicators that shared one of their constituent variables. Correlations between the latent interaction variable and its constituent latent variables as well as the other predictors were set to zero (Little et al., 2006).
